# Glycol Chitosan-Based Fluorescent Theranostic Nanoagents for Cancer Therapy

**DOI:** 10.3390/md12126038

**Published:** 2014-12-17

**Authors:** Jin-Kyu Rhee, Ok Kyu Park, Aeju Lee, Dae Hyeok Yang, Kyeongsoon Park

**Affiliations:** 1 Western Seoul Center, Korea Basic Science Institute, Seoul 120-140, Korea; E-Mail: jkrhee@kbsi.re.kr; 2 Division of Bio-imaging, Chuncheon Center, Korea Basic Science Institute, Gangwon-do 200-701, Korea; E-Mail: okpark81@kbsi.re.kr; 3 Biomedical Research Center, Korea Institute of Science and Technology, Seoul 136-791, Korea; E-Mail: fleur0903@hanmail.net; 4 Institute of Cell & Tissue Engineering, College of Medicine, The Catholic University of Korea, Seoul 137-701, Korea

**Keywords:** glycol chitosan, theranostics, nanosystem, fluorescence, cancer

## Abstract

Theranostics is an integrated nanosystem that combines therapeutics with diagnostics in attempt to develop new personalized treatments with enhanced therapeutic efficacy and safety. As a promising therapeutic paradigm with cutting-edge technologies, theranostic agents are able to simultaneously deliver therapeutic drugs and diagnostic imaging agents and also monitor the response to therapy. Polymeric nanosystems have been intensively explored for biomedical applications to diagnose and treat various cancers. In recent years, glycol chitosan-based nanoagents have been developed as dual-purpose materials for simultaneous diagnosis and therapy. They have shown great potential in cancer therapies, such as chemotherapeutics and nucleic acid and photodynamic therapies. In this review, we summarize the recent progress and potential applications of glycol chitosan-based fluorescent theranostic nanoagents for cancer treatments and discuss their possible underlying mechanisms.

## 1. Introduction

Theranostics is a system that integrates targeting, therapeutic, and diagnostic functions within an all-in-one platform [1]. Currently, diagnostic imaging and treatment processes are carried out separately in the study of the characteristics (*i.e.*, cellular phenotype, heterogenecity, *etc.*) of cancers and administration of drugs for therapy [2,3]. These separate processes require a long time period in which to evaluate drug efficacy and adjust the treatment plan accordingly, resulting in loss of opportunity for effectively treating some diseases, especially rapidly progressing cancers [4]. In contrast, integrated theranostic nanoagents can deliver diagnostic imaging agents capable of detecting and monitoring the early onset of diseases and simultaneously transport suitable therapeutic drugs over a prolonged period in order to enhance therapeutic efficacy [5]. This all-in-one theranostic approach is less time consuming and subsequently allows for a faster and more precise decision, allowing for more effective outcomes. Furthermore, theranostic technologies will radically change the way we diagnose, treat, and prevent cancer in oncology, and they show great promise in the emerging field of personalized medicine.

Advances in nanotechnology have contributed to the development of novel multifunctional nanoagents that enable specific delivery of imaging agents and therapeutic drugs to target diseased tissues for cancer imaging and therapy, and thus, they have ultimately led to the newest technology, “theranostics.” Nanotheranostics involves the application and further development of various nanoparticle systems, such as polymer conjugations, dendrimers, micelles, liposomes, metal and inorganic nanoparticles, carbon nanotubes, and polymeric nanoparticles, for sustained, controlled, and targeted co-delivery of diagnostic and therapeutic agents in order to achieve improved results and fewer side effects [6]. Since these nanoparticles have nanoscale size dimensions (10–500 nm), they can navigate through microvasculatures and across various biological barriers to preferentially accumulate in tumor tissues due to the “enhanced permeability and retention” (EPR) effects, which are the hallmark of leaky vasculatures and poor lymphatic drainage [7]. Ample functional groups and easy modification with hydrophobic segments allow these nanoparticles to be modified by imaging agents and therapeutic drugs, which can either be encapsulated by or conjugated to polymeric nanoparticles. Therefore, polymeric nanoparticles have been employed to serve as diagnostic tools, therapeutic carriers, or both [4]. Additionally, polymeric nanoparticles improve the half-lives, solubility, and stability of imaging probes and therapeutic drugs while dramatically reducing potential side effects [8,9,10]. These features make polymeric nanoparticles preferable for customized and personalized translational medicine.

**Figure 1:**
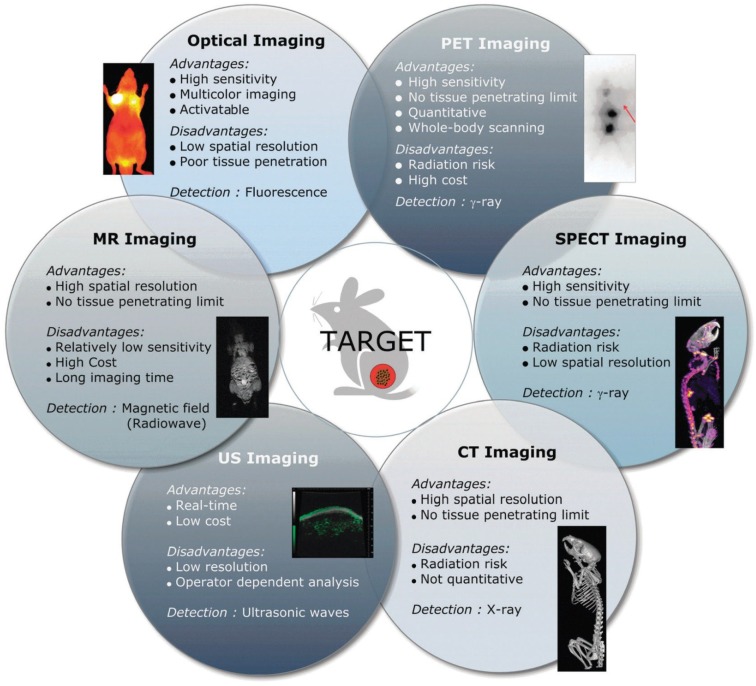
Non-invasive imaging modalities and their characteristics for biomedical applications. Permission from reference [13], Copyright © 2011, Royal Society of Chemistry.

A diverse set of non-invasive imaging modalities is employed for the detection of cancer at early stages, discovery and development of new drugs, and monitoring of drug responses by offering information about biological changes and living systems, as well as visualization of the distribution of theranostic nanoagents in real time [4,11,12]. These non-invasive imaging modalities include positron-emission tomography (PET), magnetic resonance imaging (MRI), X-ray computed tomography (CT), single photon emission computed tomography (SPECT), ultrasound (US), and optical fluorescence imaging [12]. As shown in Figure 1, each imaging modality has its own unique advantages and disadvantages regarding sensitivity, spatial resolution, cost, safety, and tissue penetration [13]. Recently, near-infrared fluorescence (NIRF) imaging techniques have been used for real-time imaging in live animals. Although poor tissue penetration must be overcome with this method when applied in clinics, the NIRF imaging system is safe, highly sensitive, and capable of multicolor imaging to monitor the fate of theranostic agents in live animals without the requirement of a local cyclotron, incontinent radionuclide-labeling step, or expensive instruments [14]. Also, an optical imaging approach in the NIR window (700–900 nm) is very useful to identify the fundamental processes at the cellular and molecular levels because the absorbance and autofluorescence of hemoglobin (the main absorber in the visible region), water, and lipids (primary absorbers of infrared light) are lower in the NIR range [15].

Chitosan, a deacetylated derivative of chitin, is a copolymer that consists of two repeating units (*i.e.*, *N*-acetyl-2-amino-2-d-glucopyranose and 2-amino-2-deoxy-d-glucopyranose) linked by a β-(1→4)-glycosidic bond [16]. Chitosan is a biocompatible, biodegradable, and low-immogenic polymer that has great potential for a wide range of biomedical applications, such as drug delivery, gene delivery, cell imaging, and as a sensor in the treatment and diagnosis of some diseases [16]. However, the poor solubility of chitosan in aqueous solutions at pH > 6.0 is a major limitation to these potential uses. Glycol chitosan (GC) is a water-soluble chitosan derivative at neutral pH due to the introduction of a hydrophilic ethylene glycol group. Over the last 10 years, GC-based polymeric nanoparticles have been developed as therapeutic carriers (*i.e.*, anticancer drugs [17,18,19], peptides [20,21], nucleic acids [22,23], and diagnostic imaging agents [24,25,26]) in oncology. This review article describes the recent progress with GC-based fluorescent theranostic nanoagents as dual-purpose agents used for simultaneous diagnosis and therapy and further discusses their possible underlying mechanisms.

## 2. Basic Components for Manufacturing Theranostic Nanoagents

**Figure 2:**
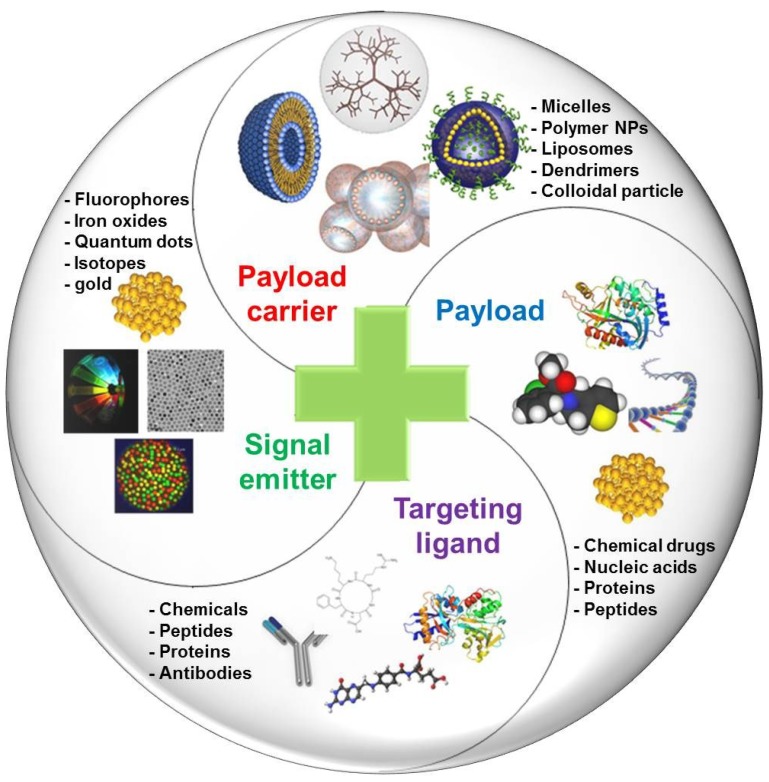
Four basic components for the design of theranostic nanoagents.

Integrated theranostic nanoagents offer multifunctional platforms for simultaneous cancer diagnosis and therapy. They provide a great deal of information for rapid disease verification and exact localization of the diseased tissues and permit the rapid delivery of therapeutic agents to the target tissues. To achieve the concept of theranostics, most theranostic nanoagents are typically designed in consideration of four basic components: signal emitter, therapeutic payload, payload carrier, and targeting ligand [4]. Representative examples of the components are shown in Figure 2 and Table 1. 

**Table 1:** Examples of four basic components for the design of theranostic nanoagents.

Components	Examples
**Signal Emitters**	**Fluorophores**: Fluorescein isothiocyanate (FITC), Alexa Fluor 488, yellow fuorescent protein (YFP), Rhodamine, tetramethylrhodamine (TRITC), cyanine 3(Cy3), red fluorescent protein (RFP), Texas Red, Cy5, Alexa Fluor 647, Cy5.5, Cy7, protoporphyrin IX (PpIX), and chlorine e6 (Ce6), *etc.*
	**Isotopes**: ^76^Br, ^124^I, ^94m^Tc, ^68^Ga, ^66^Ga, ^6^°Cu, ^64^Cu, ^89^Zr, *etc.*
	**Magnetic resonance imaging agents**: iron oxide, iron platinum, manganese, gadolinium, *etc.*
	**Other metals**: Quantom dots, silver and gold nanoparticles, *etc.*
**Payload**	**Chemical drugs**: Doxorubicin, cisplatin, paclitaxel, docetaxel, camptothecin, mitoxantrone, gemcitabin, curcumin, photosensitizers, imatinib, trastuzumab, sinutinib, cetuximab, *etc.*
	**Protein and peptide drugs**: RGD, buserelin, gonadorelin, leuprolide, triptorelin, abarelix, cetrorelix, Tumor necrosis factor-related apoptosis-inducing ligand (TRAIL), melanoma differentiation associated-7 (MDA-7), E1A, E4ORF4, VP3 (apoptin), cytokines (interleukin-2, interferon alpha-n3, interferon beta-1, inteferon alpha-2b), monoclonal antibodies (zevalin, mylotarg, bexxar, herceptin, avastin), *etc.*
	**Neucleic acids**: Plasmids, antisense oligonucleotides, ribozymes, DNAzymes, aptamers, small interfering RNA (siRNA), small hairpin RNA (shRNA), and microRNA, *etc.*
**Payload Carriers**	Macromolecular prodrugs, stealth nanoparticles, micelles, nanogels, nanocapsules, polymersomes, liposomes, dendrimers, porous silica nanoparticles, *etc.*
**Targeting Ligands**	**Angiogenic endothelial cell target**: RGD, vascular endothelial growth factor, TGN peptide, *etc.*
	**Cancer cell target**: Epidermal growth factor receptor monoclonal antibody, epidermal growth factor, human epidermal receptor 2, transferrin, A10 aptamer, As1411 aptamer, cRGD, galactose, hyaluronic acid, folic acid, glycyrrhizin, *etc.*

The signal emitter possesses certain unique optical, magnetic, or radioactive properties. Therapeutic drugs can be chemotherapeutic drugs, therapeutic proteins and peptides, or nucleic acids. The drug carrier is a kind of matrix comprised of polymeric materials. In general, intact and modified polymers have ample functional groups (such as −NH_2_, −COOH, −OH, −SH, or −N_3_, *etc.*) that offer flexibility in integrating multifunctionalities. Due to this flexibility, both the signal emitter and therapeutic drugs can be either non-covalently encapsulated in the carrier via hydrophobic and/or electrostatic interactions or covalently conjugated to the surface of the drug carrier [27,28]. Importantly, most of the synthetic and natural polymers used for biomedical applications are cleavable and biodegradable. Sometimes, the unique characteristics of tumor microenvironments, such as enzymes and pH, which would trigger drug release from a drug carrier, are used as another drug delivery approach. For example, enzymatic cleavage is highly specific for certain tumors where specific enzymes are overexpressed. Enzymatically-degradable drug delivery carriers showed a rapid drug release upon specific enzyme exposure, whereas minimal drug release occurs without enzymes. In case of non-enzymatic drug carriers, they showed the sustained delivery of therapeutic drugs at the target site. Finally, targeting ligands with high affinity to tumor cells or angiogenic endothelial cells are always covalently attached to the surface of the drug carriers [29]. Because passive targeting delivery of nanoparticles does not often operate effectively in poorly vascularized regions, an active targeting approach using a targeting ligand is required in order to increase accumulation of the nanoparticle in the disease tissue and to facilitate detection efficiency while reducing unwanted uptake by normal tissue, thus minimizing side effects. As described above, to meet the requirements for theranostics, the manufacturing processes of multifunctional theranostic nanoagents as a single system must be fairly complicated. However, theranostic nanoagents have promising advantages because they allow *in situ* imaging, targeted delivery, and controlled release of therapeutic drugs to diseases sites, as well as real-time monitoring of treatment responses.

## 3. Glycol Chitosan-Based Theranostic Nanoagents

**Figure 3:**
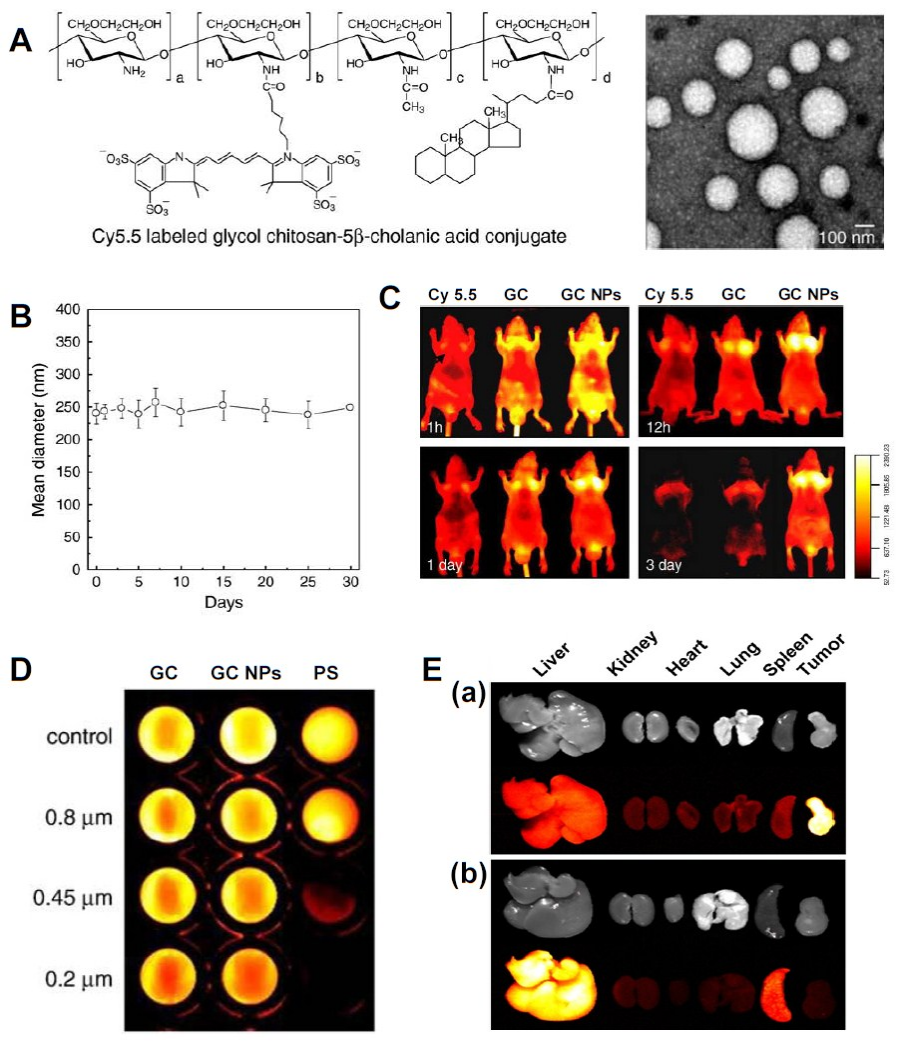
**(A**) Chemical structure of Cy5.5-labeled glycol chitosan-5β cholanic acid conjugates and TEM image; (**B**) Colloidal stability of Cy5.5-labeled GC NPs in PBS for one month; (**C**) Time-dependent tumor targeting specificity of free Cy5.5, water soluble Cy5.5-labeled glycol chitosan polymer, and Cy5.5-labeled GC NPs in SCC7 tumor-bearing mice; (**D**) Filtration test of Cy5.5-labeled glycol chitosan polymer, Cy5.5-labeled GC NPs, and Cy5.5-labeled polystyrene beads with different pore sizes; (**E**) *Ex vivo* organ distribution of (**a**) Cy5.5-labeled GC NPs and (**b**) Cy5.5-labeled polystyrene beads. Reprinted with permission from [31] and [33], Copyright © 2011 and 2010 Elsevier.

A variety of polymeric nanoparticles for drug delivery, diagnosis, and therapy have been developed. Among them, glycol chitosan (GC) has attracted interest because it is soluble in aqueous media, biocompatible, biodegradable, and has low immunogenicity [30]. Over the last 10 years, GC-based nanoparticles (GC NPs) have been intensively studied as drug delivery carriers for therapy and/or imaging agents for diagnosis. GC NPs are prepared by chemical conjugation of a hydrophilic GC polymer (250 kDa) with hydrophobic molecules. At the initial stage, GC NPs were developed as drug delivery carriers for cancer therapy. Due to their hydrophobic moieties and positive charge, GC NPs can non-covalently encapsulate hydrophobic cancer drugs [17,18,19], peptides [20], or nucleic acids [22] and still show potent therapeutic efficacy. Since 2006 when various non-invasive optical imaging modalities became widespread, optical imaging probes for the detection of various cancers (head and neck, brain, liver, colon, and metastatic cancer) were also developed by introducing a near-infrared fluorophore (Cyanine 5.5, Cy5.5) to GC NPs [24,26,31]. The constructed Cy5.5-labeled GC-5β-cholanic acid conjugates formed self-assembled nanostructures with diameters of 260 ± 30 nm and showed good colloidal stability for up to one month in PBS (pH 7.4) [24] (Figure 3A,B). To verify the superior tumor-targeting efficacy of Cy5.5-labeled GC NPs, several key factors influencing biodistribution and tumor-targeting of the nanoparticle system were investigated. Firstly, Cy5.5 (small molecule), Cy5.5-labeled GC (soluble polymer), and Cy5.5-labeled GC NPs were intravenously injected to SCC7 (squamous cell carcinoma) tumor-bearing mice. Compared to fluorescence intensities of Cy5.5 and Cy5.5-labeled GC, Cy5.5-labeled GC NPs exhibited strong fluorescence signals in the whole body and in tumor tissues for up to three days (Figure 3C), implying that the nanoparticle system has much stronger tumor-targeting characteristics than do small molecules and soluble polymer. Secondly, whole body fluorescence images of three types of Cy5.5-labeled GC NPs using different molecular weights of GC (20, 100, and 250 kDa) were performed to investigate the effects of molecular weight on tumor-targeting efficacy [24]. *In vivo* fluorescence images showed that the highest molecular weight GC NPs had a much longer circulation time in the blood stream and were more preferentially accumulated in tumor tissues than were the relatively lower molecular weight GC NPs. Thirdly, the deformability of GC NPs is also an important factor influencing tumor-targeting efficiency. As red blood cells (8 μm) of a highly flexible nature can readily pass through small micro-vessels (2.5 μm) [32], more than 95% of Cy5.5-labeled GC NPs (260 ± 30 nm) easily passed through a 200-nm pore size filter. However, 200-nm rigid Cy5.5-labeled polystyrene (PS) NPs did not easily pass through even a 450-nm pore size filter (Figure 3D). Indeed, deformable Cy5.5-labeled GC NPs showed strong NIRF signals in tumor tissues after passing through the *in vivo* filtration system of the liver and spleen [31] (Figure 3E). However, rigid and non-deformable PS NPs were primarily localized in the liver and spleen. These results suggest that the particles must be either smaller than the critical size or deformable enough to avoid *in vivo* filtration systems [33]. Finally, GC NPs have a positive surface charge and lipophilicity to facilitate rapid cellular uptake, which may improve the residence time of NPs in tumor tissues and thereby enhance the efficiency of diagnosis and therapy. These properties demonstrate the excellent tumor-targeting characteristics of GC-based theranostic NPs, making them one of the more promising of the recently developed GC-based fluorescent theranostic nanoagents that are being studied for simultaneous diagnosis and therapy.

### 3.1. Small Molecular Drug-Based Theranostic Nanoagents

As previously described, GC NPs have good colloidal stability, deformability (or flexibility), and rapid cellular uptake and are very suitable as drug delivery carriers and imaging agents. To achieve the theranostic concept in an all-in-one platform, chemotherapeutic drugs are non-covalently encapsulated in Cy5.5-labeled GC NPs. Here, Cy5.5, GC NPs, and chemotherapeutic drugs act as signal emitter, payload carrier, and payload, respectively. Using several Cy5.5-labeled GC NPs constructed by conjugation of GC with 5β-cholanic acid, deoxycholic acid, or hydrotropic oligomer, chemotherapeutic drugs, such as paclitaxel [33,34], cisplatin [35], camptothecin [36], or docetaxel [37], can be readily encapsulated into the GC NPs via hydrophobic interactions. These GC NPs can be used as a Cremophor EL (polyoxyethylated caster oil)/absolute ethanol-free formulation to solubilize extremely insoluble drugs, such as paclitaxel [33,34] and docetaxel [37]. The GC NP system can also protect the drug activity of hydrolysis-labile drugs, such as camptothecin [36]. Furthermore, hydrotropic DENA (N,N-diethylnicotinamide)-based polymeric GC NPs can enhance drug-loading content (or efficiency) by almost two-fold compared to that of GC-5β-cholanic acid NPs because hydrotropic oligomers as water-soluble compounds enhance the water solubility of sparingly soluble drugs [34,38]. With the help of *in vivo* fluorescence imaging modalities, several pieces of information were confirmed: Cy5.5-labeled GC NPs containing chemotherapeutic drugs have a long circulation time (three to seven days), show specific tumor accumulation (nearly two- to four-fold) compared to that in normal tissues [33,34,37], and exhibit dose-dependent delivery efficiency to tumors. Also, the repeated injection frequency (or intervals) of theranostic nanoagents can be determined by monitoring the NIRF signal in tumor tissues. Therefore, based on the obtained important information driven by NIRF whole and *ex vivo* images, the therapeutic efficacies of the nanoagents can be greatly maximized. This evidence suggests that the use of theranostic nanoagents will play an important role in attaining more specific information, such as circulation time, biodistribution, nanoparticle injection intervals and dosages, and therapeutic response monitoring, allowing for optimized treatment protocols.

Typically, when researchers develop theranostic nanoagents, they make an effort to produce optimized systems with high targeting efficiency. However, bare nanoparticles and drug-loaded nanoparticles have quite different biodistributions and tumor-targeting characteristics [39]. In fact, the *in vivo* fate of a nanoparticle system can be altered because the physicochemical properties of nanoparticle systems, such as particle size, colloidal stability, and deformability, can be different before and after drug encapsulation into nanoparticle systems. Therefore, researchers should perform *in vivo* studies with a single platform containing both signal emitter and payload in order to obtain more accurate and precise information regarding theranostic nanosystems.

### 3.2. siRNA-Based Theranostic Glycol Chitosan Nanoagents

Nucleic acid therapy is promising for the treatment of various diseases, such as Alzheimer’s disease, cancer, adenosine deaminase deficiency, and cystic fibrosis [40]. Current and potential therapeutic nucleic acids include plasmids, antisense oligonucleotides, ribozymes, DNAzymes, aptamers, and small interfering RNA (siRNA). These therapeutic nucleic acids can alter gene expression at the transcriptional or post-transcriptional level and may be effective in treating cancer and cardiovascular and inflammatory diseases. Although they are powerful therapeutic agents for the treatment of diseases, there are still obstacles to be overcome. Naked nucleic acids are highly susceptible to enzymatic degradation. Also, large and negatively-charged nucleic acids are not easily internalized by cells due to the difficult in crossing negatively-charged cell membranes. Thus, naked nucleic acid delivery requires direct injection of nucleic acids to the disease areas via physical methods, such as electroporation, gene gun, or ultrasound [41]. However, systemic delivery of naked nucleic acids is quite ineffective due to the minimal amount of intact polynucleotide reaching diseased sites because of the degradation by nucleases within the physiological fluids. Also, the direct injection approach limits efficient delivery only to anatomically accessible target sites.

Recently, among various nucleic acids, siRNA has attracted a great deal of attention as a potential therapeutic agent due to its highly sequence-specific gene silencing ability and generality in therapeutic targets. The difficulty of systemic siRNA is that its delivery is severely hindered in clinical applications due to its susceptibility to degradation by nuclease. To achieve efficient systemic delivery of therapeutic siRNA, many researchers have focused on the development of nonviral vehicles, which are nontoxic and less immunostimulatory and can have more versatile modifications for carrying different types of drug payloads. GC NPs have been tested for systemic delivery of siRNA; however, the electrostatic interaction between positively-charged GC NPs and negatively-charged siRNA is too weak to form condensed and stable siRNA/GC NP vehicles. To obtain vehicles that are more condensed and stable, GC-5β-cholanic acid and PEI-5β-cholanic acid were mixed together at a 1:1 weight ratio to yield GC-PEI NPs [23]. RFP-siRNA and GC-PEI NPs at a weight ratio of 1:5 formed more condensed and stable nano complexes (250 nm) with nearly 100% loading efficiency due to the increased positive charge caused by mixing GC NPs with PEI NPs. The RFP-siRNA/GC-PEI NPs successfully protected encapsulated RFP-siRNA from degradation by RNase A. Also, GC-PEI NPs effectively delivered RFP-siRNA to the cell cytoplasm and exerted remarkable silencing effects by suppressing RFP expression in RFP-expressing B16F10 cells. *In vivo* fluorescence images revealed that the intravenously injected Cy5.5-labeled RFP-siRNA/GC-PEI NPs were highly accumulated in tumor tissues. Due to effective delivery of RFP-siRNA into tumor tissues, the RFP signal intensity of RFP-B16F10 tumors treated with RFP-siRNA/GC-PEI NPs was 4.8-fold lower than that of the free siRNA group.

**Figure 4:**
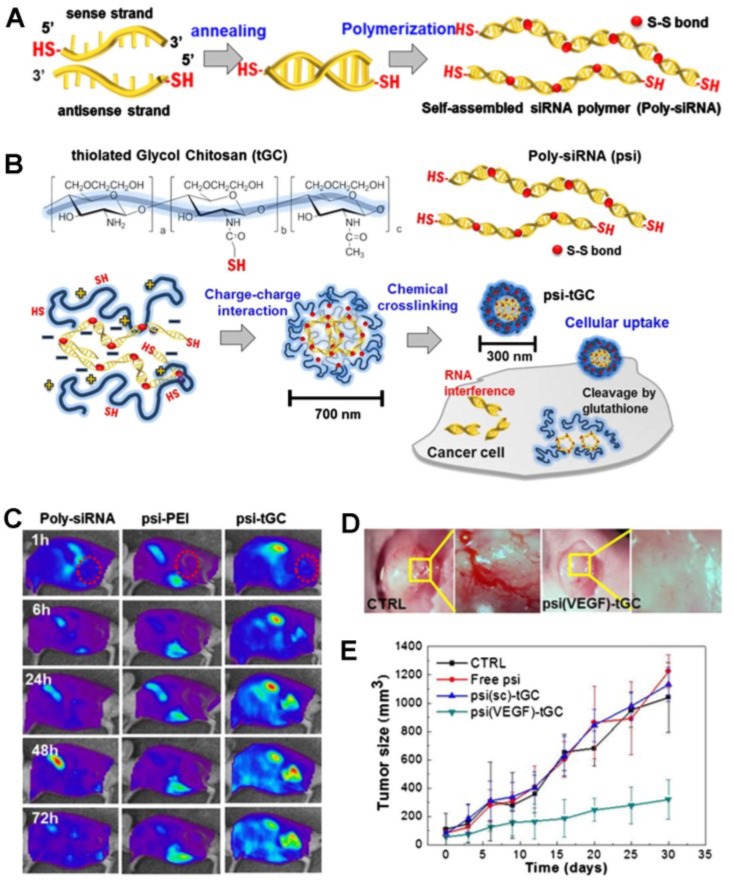
(**A**) Scheme of polymerized siRNA (poly-siRNA) synthesis. Reprinted with permission from [42], Copyright © 2009 Elsevier B.V.; (**B**) Preparation of poly-siRNA/tGC nanoparticles for siRNA delivery; (**C**) *In vivo* real-time NIRF imaging of poly-siRNA/tGC in SCC7 tumor-bearing mice after i.v. injection of FPR675-labeled nanoparticles; (**D**) Inhibition of blood vessel formation by poly-siRNA (VEGF)/tGC; (**E**) Antitumor effects of control, poly-siRNA, poly-siRNA (scramble)/tGC, and poly-siRNA (VEGF)/tGC. Reprinted with permission from [43], Copyright © 2012 WILEY-VCH Verlag.

Kim and his colleagues synthesized a novel alternative siRNA, polymerized siRNA (poly-siRNA), to enhance the stability of the siRNA [42]. To polymerize siRNA, dithiol-modified siRNA (RFP- or VEGF-specific siRNA) bearing thiol groups at the 5′-ends of both sense and anti-sense strands were reacted in the presence of *N*,*N*,*N*′,*N*′-tetramethyl-azodicarboxamide (Figure 4A). The polymerized siRNA consisted of a broad range of base pairs (50–300 bps and greater than 300 bps) through disulfide-polymerization of thiol groups at both ends of the modified siRNA. This polymerized siRNA can be converted to original mono-siRNA in the presence of a reducing agent, such as dithiothreitol (DTT) or under reductive conditions as exist in the cell cytosol. The *in vitro* serum stability test showed that mono-siRNA was degraded within 1 h, whereas most of the poly-siRNA was degraded within 12 h, suggesting that poly-siRNA is much more stable and less degradable by serum nuclease compared to mono-siRNA [43]. Although poly-siRNA is more stable than mono-siRNA, the degradation of poly-siRNA is still very susceptible to enzymatic degradation in *in vivo* physiological fluids. To overcome this problem, Kim and his colleagues synthesized a new systemic poly-siRNA delivery carrier, thiolated GC (tGC) polymer, using sulfosuccinimidyl-6-[3′(2-pyridyldithio)-propionamido] hexanoate (Sulfo-LC-SPDP) and DTT (Figure 4B) [43]. The tGC polymer formed stable nanostructures with poly-siRNA (RFP or VEGF) through enhanced electrostatic interaction and a self-crosslinking mechanism, leading to increased serum stability up to 24 h. The poly-siRNA/tGC (psi-tGC) NPs rapidly internalized and localized in the cytosol within 1 h and then showed efficient RFP gene silencing. After intravenous injection, FPR 675-labeled poly-siRNA-tGC was preferentially accumulated in tumor tissues compared to naked FPR 675-labeled poly-siRNA or FPR 675-labeled-psi/PEI polyplexes, suggesting higher tumor selectivity of poly-siRNA-tGC NPs (Figure 4C). *In vivo* fluorescence images and therapeutic tests demonstrated that poly-siRNA(RFP)-tGC showed effective RFP gene silencing *in vivo*, and poly-siRNA(VEGF)-tGC NPs also significantly inhibited neovascularization via effective and specific VEGF gene silencing, leading to successful tumor suppression (Figure 4D,E). These results support the theory that the modified GC derivatives are suitable vehicles for siRNA delivery. However, more optimized treatment protocols for safe and effective systemic siRNA delivery carriers are still required for a wide range of clinical applications of siRNA.

### 3.3. Photosensitizer-Based Theranostic Nanoagents

Photodynamic therapy (PDT) is a relatively new type of treatment that is attracting interest as a potential cancer treatment. PDT is a nonthermal photochemical reaction, which requires the simultaneous presence of a photosensitizing drug, oxygen, and a special type of light [44]. The photosensitizing drug alone is harmless in the absence of light and oxygen. The administered photosensitizing drug is only activated by irradiation of light to generate reactive oxygen species (ROS), including singlet oxygen (^1^O_2_), hydroxyl radicals (·OH), and superoxide (O^2−^), which subsequently trigger lethal oxidative stress and membrane damage in the treated cells, resulting in apoptosis, necrosis, or autophagy at the area of light exposure [44,45,46,47,48]. In addition, photosensitizing drugs show unique luminescent properties that can be useful as fluorescence imaging agents to track, visualize, and quantify the photosensitizing drugs in diseased tissues [48]. These dual features of PS, including signal emission and payload (drug) delivery, allow photosensitizing agents to act as theranostic agents for simultaneous diagnosis and image-guided therapy.

Currently, porphyrins and their derivatives are widely used as photosensitizers (PSs) in PDT. Some of these substances are already approved for use as drugs in malignant [49] or nonmalignant disease therapies [50]. However, many first- and second-generation PSs are limited in animal studies and clinical use because of non-specific skin phototoxicity, poor water solubility at physiological pH, and inefficient delivery to target tumor tissues in cancer treatment [48,51,52]. However, these limitations can be overcome by using nanoscale drug carriers. Hydrophobic PS can be encapsulated in nanoparticles or directly chemically conjugated with water-soluble polymers to enhance solubility and dispersion in an aqueous solution. Also, PS-loaded or -conjugated nanoparticles are more preferentially accumulated in tumor tissues through EPR effects [53]. The Kwon group developed protoporphyrin IX (PpIX)- and chlorin e6 (Ce6)-loaded GC NPs that are well dispersed in aqueous solution and form stable nano-structures with an average diameter around 300 nm [54,55]. These NPs also showed time-dependent release of PpIX or Ce6 from GC NPs and efficient photodynamic therapy in *in vitro* and *in vivo* studies. However, drugs physically loaded in nanoparticles showed burst drug release from nanoparticles during circulation *in vivo* [54,55]. This undesirable instability of PpIX- or Ce6-loaded GC NPs resulted in low drug delivery efficiency and decreased therapeutic effect at tumor sites as well as unintended damage to normal tissues [56]. These problems of PS-loaded nanoparticles can be overcome by chemical conjugation of PpIX or Ce6 with water soluble GC. PpIX- or Ce6-conjugated GC also formed stable and self-assembled nanoscale particles (about 250–300 nm) [55,57]. Because PpIX- or Ce6-conjugated GC NPs did not exhibit burst release from NPs due to chemical conjugation with GC, they showed less photo-toxicity compared to PpIX- or Ce6-loaded GC NPs in an *in vitro* study. However, *in vivo* imaging studies showed that they had a prolonged circulation time and accumulated more specifically in the tumor, resulting in better therapeutic efficacy. For example, Ce6-conjugated GC NPs showed a dramatic decrease of tumor volume (about 160 mm^3^) at 20 day post-treatment, which was significantly smaller than that of Ce6-loaded GC NPs-treated mice (about 560 mm^3^) [55]. These results suggest that the factors of burst drug release and stability of NPs *in vivo* should be considered when constructing photoactivatable theranostic nanoagents for PDT.

**Figure 5:**
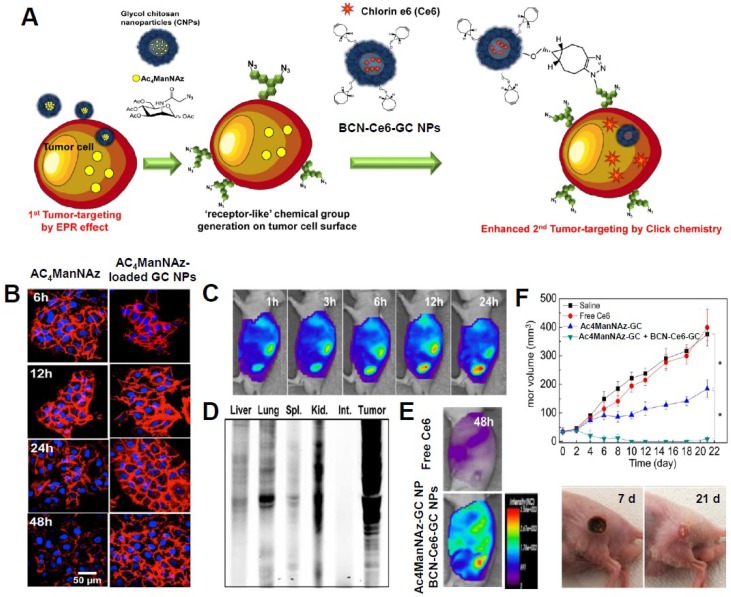
(**A**) Schematic illustration of the two-step *in vivo* tumor targeting strategy for nanoparticles via metabolic glycoengineering and click chemistry; (**B**) Time-dependent lifetime of azide groups generated by free Ac_4_ManNAz and Ac_4_ManNAz-loaded GC NPs; (**C**) Intravenous injection of Ac_4_ManNAz-loaded GC NPs and metabolic glycoengineering on tumor tissue *in vivo*; (**D**) Western blot analysis of major organs and tumor tissue after i.v. injection of AC_4_ManNAz-loaded GC NPs; (**E**) Tumor targeted image of BCN-Ce6-GC NPs in tumor bearing mice after pretreatment of Ac_4_ManNAz-GC NPs; (**F**) *In vivo* photodynamic therapy in tumor bearing mice and tumor images of mice treated with BCN-Ce6-GC NPs and Ac_4_ManNAz-GC NPs at day seven and 21. Reprinted with permission from [68], Copyright © 2014 American Chemical Society.

The bioorthogonal chemical reporter strategy is a method for labeling and visualizing biomolecules *in vivo* without the requirement of genetic manipulation [58,59]. In this approach, metabolic labeling of the cell with azides primes the target biomolecule to be visualized by covalent attachment of an imaging probe. As a chemical reporter, azide is the most widely used because of its small size, metabolic stability, and lack of reactivity with natural biofunctionality [58,60]. The reaction of azide-alkyne cycloaddition forms an azide and a terminal alkyne (called “click chemistry”), which involves the use of a Cu catalyst [61,62]. Among various alkyne reagents, cyclooctynes react with azides without the use of copper, referred to as “Cu-free click chemistry,” to achieve bioorthogonal labeling [63]. Copper-free click chemistry has been widely applied in biological and biomedical fields, such as in the labeling of proteins, nucleotides, or cells; in the analysis of metabolic pathways; and for the surface modification of nanoagents [64,65,66,67]. Recently, Lee *et al.* reported a novel two-step photoactivatable theranostic strategy *in vivo* via metabolic glycoengineering and Cu-free click chemistry (Figure 5A) [68]. To achieve this approach, they prepared two different nanoagents as follows. Firstly, for generation of azide groups on tumor tissues, the precursor (tetraacetylated *N*-azidoacetyl-d-mannosamine, Ac_4_ManNAz) was loaded into GC NPs with an amphiphilic structure via hydrophobic interactions. When Ac_4_ManNAz-loaded GC NPs were added to tumor cells or tumor-bearing mice, azide groups were site-specifically generated on tumor cells or tissues by metabolic glycoengineering after effective delivery and rapid uptake of Ac_4_ManNAz-loaded GC NPs into tumor cells. An *in vitro* cell study showed that Ac_4_ManNAz-loaded GC NPs enabled a longer lifetime for the azide groups on cells compared to free Ac_4_ManNAz because of the sustained release of Ac_4_ManNAz from GC NPs (Figure 5B). Furthermore, Ac_4_ManNAz-loaded GC NPs successfully generated large amounts of azide groups irrespective of the kind of tumor cells targeted (KB, A549, U87MG, MCF7, MDA-MD-468, and MDA-MD-436). Immunohistochemistry and fluorescence images also demonstrated that Ac_4_ManNAz-loaded GC NPs with a longer circulation time and a higher tumor-targeting efficiency effectively delivered Ac_4_ManNAz, resulting in much greater generation of the azide group on tumor tissues compared to normal tissues (*i.e.*, liver, lung, spleen, and kidney) (Figure 5C,D). Secondly, for specific delivery of photoactivatable agents to azide groups generated on tumor tissues by copper-free click chemistry, bicycle [6.1.0] nonyne (BCN)-PEG-NHS and Ce6 were conjugated with glycol chitosan polymer to obtain BCN-Ce6-GC NPs. Through copper-free click chemistry, BCN-Ce6-GC NPs specifically bind with azide groups generated on tumor cell membranes by Ac_4_ManNAz-loaded GC NPs. Compared to free Ce6 and Ce6-GC NPs, BCN-Ce6-GC NPs were more accumulated in tumor regions after azide generation caused by pretreatment with Ac_4_ManNAz-loaded GC NPs (Figure 5E). *In vivo* photodynamic therapy demonstrated that a two-step strategy using Ac_4_ManNAz-loaded GC NPs and BCN-Ce6-GC NPs showed significant black scab generation and effective tumor destruction after laser irradiation compared to use of free Ce6 or BCN-Ce6-GC NPs alone (Figure 5F). This two-step theranostic approach has great potential for the enhancement of the tumor-targeting ability of theranostic agents and their therapeutic effects in cancer therapy. However, further optimized conditions are required to more specifically generate azide groups on target-diseased sites.

Tumor tissues have a more acidic microenvironment due to lactic acid production in hypoxic areas. Indeed, solid tumors with a pH ranging from 5.8 to 7.7 are on average 0.5 units lower than the pH of normal tissues. Thus, the use of different pH environments has been a promising avenue for cancer imaging and therapy. Recently, Park *et al.* developed a photoactivatible theranostic nanoagent that quickly switches into an aggressive molecule for tumor imaging and destruction within the acidic environment of the tumor [69]. The smart pH-sensitive photoactivatable theranostic system consists of a GC backbone, a functional 3-diethylaminopropyl isothiocyanate (DEAP, pH sensitive moiety), a Ce6 block (photosensitizer), and polyethylene glycol (PEG). This theranostic nanoagent includes an intelligent switch from self-assembly (*i.e.*, self-quenched state of photosensitizer) at physiological pH 7.4 into extended random molecules (*i.e.*, dequenched state for ROS production) at the extracellular acidic pH. At physiological pH 7.4, it forms a self-assembled molecule, 150 nm in diameter, and shows no singlet-oxygen production or noticeable cell death. However, when exposed to extracellular acidic pH (pH 6.8 or 6.4), it changes to the dequenched state, thereby emitting a strong NIRF signal and singlet-oxygen generation, resulting in higher phototoxicity and efficient tumor destruction of HeLa cells. This pH-sensitive photoactivatable smart system enables targeted high-dose cancer therapy while ensuring the safety of normal tissues.

### 3.4. Fullerene-Based Theranostic Nanoagents

Fullerene (C_60_) is a soccer ball-shaped structure with 12 pentagons (due to C_5_-C_5_ single bonds) and 20 hexagons (C_5_-C_6_ double bonds). Since the discovery of C_60_ in 1985, fullerene has attracted much attention and has been viewed as having great potential for a variety of applications. Due to its unique chemical structure, C_60_ possesses interesting photo-physical properties and generates ROS by exposure to visible light [70] and thereby can be used as a potentially strong photoactivatable agent for PDT in biological systems [44]. However, due to its inherent extreme hydrophobicity and innate tendency to aggregate in water and biological media, C_60_ has inefficient photoactivity and is less promising for application in photoactivatable drugs in biomedicine [44]. To overcome this shortcoming of C_60_, it was chemically conjugated to polysaccharides, such as GC and hyaluronic acid [71,72,73]. Unlike pristine C_60_ molecules, which rapidly aggregated within 5 min, GC-C_60_ conjugates formed self-organized nanoparticles (approximately 10-23 nm in diameter) in PBS and were stable for more than one month without any precipitation [71]. Solubilization of C_60_ seemed to improve the light-sensitization of C_60_ molecules. When illuminated with a 670-nm laser source, GC-C_60_ conjugates generated singlet oxygen and significantly induced KB cell death, whereas free C_60_ conjugates did not. More recently, Kim *et al.* developed an endosomal pH-activated GC-fullerene derivative for PDT that was prepared using a simple two-step chemical reaction of (i) 2,3-dimethylmaleic acid (DMA) to free amine groups of GC and (ii) free hydroxyl groups of GC-DMA to π-π carbon bonds of C_60_ [73]. The GC-DMA-C_60_ conjugates formed self-assembled multi-nanogel aggregates (283 nm) at pH 7.4 due to the electrostatic interactions between pendant DMA groups and the residual free amine groups of GC. During light illumination at 670 nm, GC-DMA-C_60_ nanogels showed less generation of singlet oxygen and less cytotoxicity of KB cells at pH 7.4 owing to an increased photo-interference effect between C_60_ molecules close packed in multi-nanogel aggregates. However, these multi-nanogel aggregates were divided into single nanogel parts (approximately 46 nm) at endosomal pH 5.0 due to the reduction of electrostatic interactions resulting from cleavage of the DMA blocks [74]. In particular, singlet oxygen generation and phototoxicity were significantly increased at endosomal pH 5.0. Another important consideration is that the solubilized GC-fullerene derivatives enable photo-luminescent tumor imaging without labeling of any fluorophores or isotopes [71,72,73]. Indeed, *in vivo* fluorescence images demonstrated that GC-C_60_- or GC-DMA-C_60_-emitting fluorescence signals accumulated in tumor tissues in KB tumor-bearing mice. These solubilized fullerene derivatives are useful and promising for PDT. However, the fluorescence intensities of fullerene derivatives seem to be weaker than those of NIR fluorophores and other photosensitizers. These concerns should be further investigated to achieve high-resolution in fluorescence imaging.

## 4. Conclusions

This review article discusses the recent progress of GC-based fluorescent theranostic nanoagents for simultaneous diagnosis and therapy in cancer treatments. Due to their biocompatible, biodegradable, and low-immunogenic properties, GC and its derivatives have been extensively studied for use in a wide range of biomedical applications. In particular, GC polymer has a large number of amine groups on its GC backbone and so can be covalently or non-covalently modified with hydrophobic molecules (bile acid analogs), drugs (chemotherapeutic small molecular drugs, nucleic acids, photosensitizers, or fullerenes), signal emitters (NIR fluorophores, photosensitizers, or fullerenes), and other imaging tracers (isotopes) in order to yield theranostic nanosystems. These GC-based fluorescent theranostic NPs showed good colloidal stability for extended circulation in the blood stream, excellent deformability to avoid *in vivo* filtration by the liver or spleen, and/or rapid cellular uptake characteristics to facilitate delivery of theranostic agents to target sites. Due to these features, GC-based theranostic nanosystems have excellent tumor-targeting characteristics, leading to effective therapeutic results. Therefore, GC-based theranostic nanosystems will play important roles in future biomedical applications and personalized medicine. In almost all works concerning chitosan applications, chitosan and its derivatives are non-toxic, biologically compatible materials, and thus suitable for the drug delivery carriers. Sometimes, the issue for their biocompatibilities is completely disregarded due to the statement on chitosan approval by the American Food and Drug Administration (FDA) as a wound dressing material [75]. However, their biocompatibility must be addressed and considered separately, requiring specific testing in the particular conditions expected for its administration because each particular case may be quite different in several different structures, formulation, and carriers in varied conditions [76]. The manufacturing processes also needed to meet the requirements for simultaneous diagnosis and therapy of theranostic nanosystems in a single platform are generally complicated. Due to the increased complexity, the technical challenges, such as cost, colloidal stability, and reproducibility, must be considered. Future improvements should be focused on the development of innovative strategies to overcome these technical challenges.
